# The existence of cranial bone flap displacement during brain radiotherapy

**DOI:** 10.1016/j.tipsro.2024.100250

**Published:** 2024-04-14

**Authors:** Nikolina E. Birimac, Yves C.P. Willems, Catharina M.L. Zegers, Femke Vaassen, David Hofstede, Inge Compter, Jaap Jaspers, Alejandra Méndez Romero, Martinus P.G. Broen, Ans Swinnen, Olaf E.M.G. Schijns, Mirko Unipan, Ruud M. Houben, Wouter van Elmpt, Daniëlle B.P. Eekers

**Affiliations:** a Department of Radiation Oncology (Maastro), GROW School for Oncology and Reproduction, Maastricht University Medical Center, Maastricht, the Netherlands; b Department of Radiotherapy, Erasmus MC Cancer Institute, Erasmus University Medical Center, Rotterdam, the Netherlands; c Department of Neurology, Maastricht University Medical Center, Maastricht, the Netherlands; d Department of Neurosurgery, Maastricht University Medical Centre, Maastricht, the Netherlands; e Academic Center for Epileptology, Maastricht University Medical Centre and Kempenhaeghe, Maastricht, the Netherlands; f School for Mental Health and Neuroscience (MHeNS), University Maastricht (UM), Maastricht, the Netherlands; g Department of Radiation Oncology, Radboud University Medical Centre, the Netherlands

## Abstract

This retrospective study examined bone flap displacement during radiotherapy in 25 post-operative brain tumour patients. Though never exceeding 2.5 mm, the sheer frequency of displacement highlights the need for future research on larger populations to validate its presence and assess the potential clinical impact on planning tumour volume margins.

## Highlights

• Retrospective study of 25-post-operative brain tumour patients.
• First documented literature recording the displacement of cranial bone flaps over the course of radiation treatment.
• Displacement, though never exceeding 2.5 mm, was found to have a high frequency in all patients.
• Bone flaps found not to remain a fixed entity as is assumed when cropping the CTV to the bone.
• Provides insight for future research to focus on determining the impact of displacement on planning treatment margins.

## Introduction

**Fig. 1:**
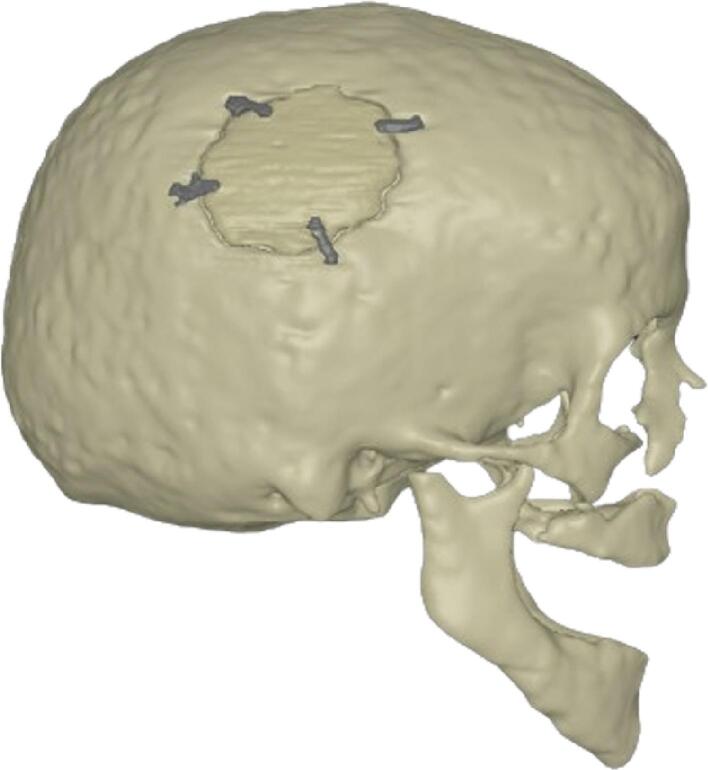
3D rendition of a bone flap, affixed to the skull with four fixation plates, from this study.

A common treatment approach for primary brain neoplasms in adults is maximum safe resection followed by radiotherapy with or without chemotherapy [1], [2], [3], [4]. Following surgical resection, patients are left with a cranial bone flap (Fig. 1), which could impact the next treatment stage as most delineated radiotherapy target volumes are often determined relative to the skull bone [5], [6], [7], [8].

**Fig. 2:**
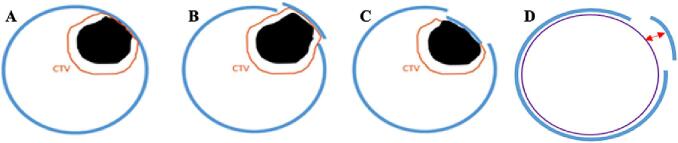
Theorised impacts of bone flap displacement on planning margins. A) Clinical Target Volume (CTV; orange) defined in relation to skull bone, B) potential change in CTV with extracranial displacement, C) potential change in CTV with intracranial displacement, D) and measurement (red arrow) between the brain OAR and bone flap. Resection cavity; black, skull bone; blue, brain OAR; purple. (For interpretation of the references to colour in this figure legend, the reader is referred to the web version of this article.)

Given that the Clinical Target Volume (CTV) is cropped to anatomical boundaries like the skull, bone flap displacement could alter the position of the boundary to which the CTV is defined. Consequently, this could potentially impact the size of the CTV and subsequent Planning Target Volume (PTV), a target volume representing the CTV with an additional enlarged margin to account for uncertainties/changes between the planning and treatment stages related to patient set-up [7]. Fig. 2 visually demonstrates the theorised impacts of extracranial or intracranial displacement of the bone flap on the resection cavity and subsequent CTV margin. As effective treatment relies on the adequate dosage of the CTV via treatment of the PTV, the potential impact of bone flap displacement on this target area could have clinical significance. Despite this potential effect on treatment planning and outcomes, there is limited research regarding displacement, resulting in it being an uncontrolled phenomenon. Because of this limited documentation, the precise impact of bone flap displacement on treatment coverage and outcomes remains unknown; however, there are two theorised scenarios. On the one hand, the movement is minimal and the dosimetric effect on the CTV will likely be minimal due to the used PTV margins. On the other hand, if bone flap displacement is significant, it can result in inadequate dose delivery to the CTV, in which case accommodation for the anatomical displacement by adding an internal target volume (ITV) is warranted to maintain sufficient dose coverage.

Considering the unknowns regarding the existence of bone flap displacement and its potential to impact treatment planning and outcomes, this study aims to investigate the displacement by quantifying its occurrence and evaluating three potential risk factors for significant movement: 1) Time of day; many patients in this institute anecdotally reported experiencing more movement and discomfort during evening treatments than morning treatments, 2) Bone flap fixation technique; it is hypothesised that the use of more fixation plates reduces displacement compared to using sutures or fewer fixation plates; and 3) Time interval between resection and first treatment fraction; displacement may fluctuate over time—immediately post-op, oedema and swelling restrict bone flap displacement whilst bony consolidation fixates the bone flap and limits displacement long-term, creating a potential interim window where displacement is more likely.

## Materials and methods

### Patient population

For this study, 25 post-operative brain tumour patients irradiated between October 2019 and April 2020 at Maastricht Radiation Oncology (Maastro), Maastricht, the Netherlands were retrospectively analysed for this study. Inclusion criteria were daily cone-beam CT (CBCT) scans performed before every fraction, a visible bone flap on all CBCTs, and an initial planning CT. The number of CBCT scans available per patient ranged between 28 and 33, with the exact amount depending on the tumour type of the patient and the corresponding amount of treatment fractions. An overview of the patient characteristics can be found in Table 1.

**Table 1:** Patient characteristics.

Characteristic	Median or value (range)
Treatment type	
Intensity modulated proton therapy	18
Volumetric modulated arc therapy (photon radiation)	7
Age at first fraction (years)	48.4 (24.2 – 79.6)
Tumour type (no. patients)	
Glioma (WHO II-IV)	20
Meningioma	4
Craniopharyngioma	1
Time between surgery & 1st fraction (days)	157 (30–1534)
<0.5 year (no. patients)	14
0.5–1 year (no. patients)	4
1–3 years (no. patients)	4
>3 years (no. patients)	3
Fixation technique	4
Sutures	4
1–3 fixation plates	11
4–5 fixation plates	10
CBCTs per time of day category	
Morning (07.30 – 12.29)	406
Afternoon (12.30 – 17.29)	236
Evening (17.30 – 22.29)	85

### Bone flap displacement measurements

To assess bone flap displacement over the course of radiotherapy treatments, the initial planning CT and CBCTs per patient were rigidly fused in RayStation (RaySearch Laboratories AB, Stockholm, Sweden) using an in-built automatic image registration tool that was then manually assessed and corrected for accuracy. Displacement was defined as changes in the distance measured between the bone flap and a delineated organ at risk (OAR) brain structure on the CBCTs in relation to the same measurement on the original planning CT. The brain OAR was created using an auto-contouring function in Eclipse (Varian Medical Systems Inc, Palo Alto, California, USA) and adjusted to conform with EPTN contouring atlas guidelines [9].

To ensure accurate and fair comparison, all measurements per patient were made at the same point on all scans, denoted by spatial imaging coordinates (X, Y, Z values alongside the imaging plane). Initially, to determine these points, the latest CBCT scan was compared to the original planning CT per patient to find a recognisable spot along the brain OAR border and the bone flap where there was most visual movement of the bone flap. The last CBCT was selected to establish these imaging coordinates with the rationale that the largest difference in bone flap positioning can be expected on it owing to the largest time interval between the planning CT and a CBCT. The point was also chosen so that it could be reliably determined on all CBCTs; once the imaging coordinates were positioned on the planning CT, the corresponding location was simultaneously identified on each patient’s following CBCT scans via the rigid registration between each CBCT and the original planning CT. At each patient’s individual imaging coordinates on all CBCTs, measurements in millimetres were manually made using a ruler measurement tool in the Eclipse software to determine the distance between the bone flap and the brain OAR. From these measurements, the initial distance between the bone flap and brain OAR on the planning CT was subtracted, resulting in an overall displacement value per CBCT. Extracranial displacement was denoted as a positive value (>0 mm), while intracranial displacement was expressed as a negative value (<0 mm). All patients’ measurements were initially made by a single individual (YW). To assess the overall reliability of YW’s measurements, two individuals (NB, DH) independently repeated the measurements for two patients.

### Statistical analysis

All tests were performed using IBM SPSS Statistics for Windows (version 26, IBM Corporation, Armonk, New York, USA). A p-value of < 0.05 was considered statistically significant.

To investigate the influence of time of day, every CBCT scan was categorised according to time of irradiation (morning (7.30 – 12.29), afternoon (12.30 – 17.29), and evening (17.30 – 22.29)), and the displacement between these groups was compared using a repeated-measures ANOVA after Mauchly’s test revealed that the assumption of sphericity had been met.

To investigate the relationship between bone flap fixation technique and displacement, patients were grouped into either having sutures, 1–3 fixation plates, or 4–5 fixation plates and these groups were analysed using a Kruskal-Wallis test with post-hoc pairwise Mann-Whitney U tests and Bonferroni correction.

To explore the relationship of time elapsed between surgical resection and first treatment fraction with displacement, a Spearman’s rank correlation co-efficient was performed. Additionally, the patients were grouped as < 0.5 years between surgery and first treatment fraction, 0.5–1 year, 1–3 years, or > 3 years, and these groups were then compared with a one-way ANOVA.

To assess reliability of the measured displacement, a two-way mixed effects Interclass Correlation Coefficient (ICC) was performed in SPSS on three individuals’ measurements (YW, NB, DH) for two patients.

## Results

### Bone flap displacement

**Fig. 3:**
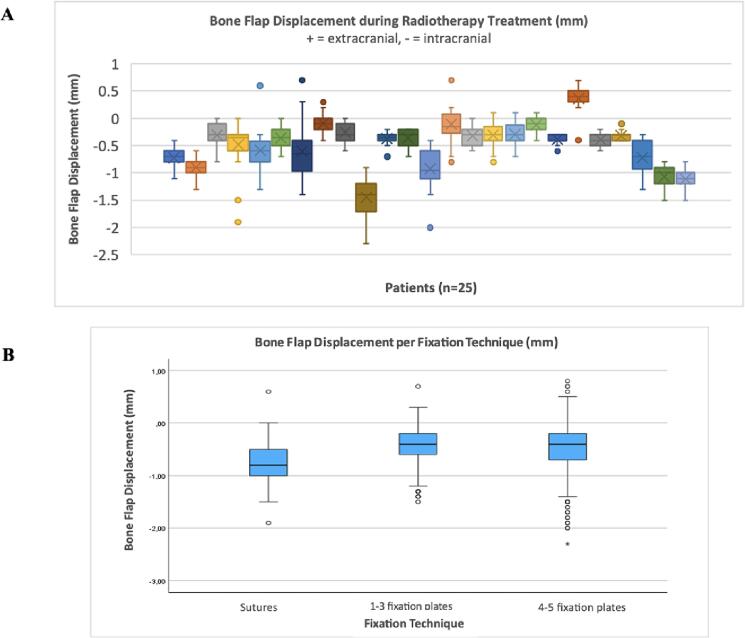
Bone Flap displacement in radiotherapy. (A) Boxplots depicting the average bone flap displacement (mm) between planning CT and CBCTs per patient over a 5–7-week radiation treatment course. (B) Boxplots of average displacement between the different fixation technique groups: sutures, 1–3 fixation plates, 4–5 fixation plates.

With one displacement measurement between each patient’s planning CT and subsequent CBCTs, a range of 28–33 measurements of displacement was recorded per patient depending on the total number of CBCTs present for each patient. With a total of 727 CBCT scans present across all patients, there was a total of 727 displacement measurements in this study upon which all subsequent results are based. The average bone flap displacement measured across all CBCTs was −0.49 mm. From all observed extracranial displacement values (54/727; 7 %), the average was 0.34 mm. From all observed intracranial displacement values (644/727; 89 %), the average was −0.58 mm. There was no observable displacement in 29 scans (4 %). The maximum recorded extracranial and intracranial displacement value was 0.8 mm and − 2.3 mm, respectively. The maximum spread of movement within a single patient was 2.1 mm (-1.4 to + 0.7 mm). Fig. 3.A depicts a box plot of the average bone flap displacements across all patients.

The reliability of the displacement measurements made were tested for two patients. These two patients had 28 CBCT scans each, resulting in 28 measurements of displacement for each patient. Each measurement was repeated by each of the three observers, yielding a total of 84 measurements per patient (three measurements of displacement per CBCT). For the two patients with repeated measurements, ICC values of 0.92 (95 % CI: 0.86 – 0.96) and 0.97 (95 % CI: 0.95 – 0.99) were yielded, respectively, indicating an overall high reliability and consistency. The high reliability observed in the repeated measurements for the two patients indicates that YW's measurements for the remaining patients likely has a similar level of reliability and accuracy in measuring displacement.

### Risk factors

The comparison of bone flap fixation technique with bone flap displacement resulted in a statistically significant difference (p < 0.001). Post-hoc analysis showed statistically significant differences between the group with sutures versus the group with 1–3 fixation plates and between the group with sutures versus 4–5 fixation plates (p < 0.001, p < 0.001 post-Bonferroni correction, respectively). Bone flap displacement was slightly increased in the group with sutures compared to those with fixation plates, as demonstrated by the 95 % confidence interval (Fig. 3.B): −0.83 mm to −0.70 mm (sutures), −0.47 mm to −0.39 mm (1–3 fixation plates), and −0.50 mm to −0.38 mm (4–5 fixation plates).

No significant relation was established between the brain surgery and first treatment fraction time interval and bone flap displacement during treatment. Additionally, no significant differences were detected between the grouped time periods with one-way ANOVA.

The effect of time of day on bone flap displacement was also found to not be significant (p = 0.608).

## Discussion

This study aimed to quantify bone flap displacement during brain radiotherapy in post tumour resection patients. The consistency of displacement, especially intracranial, observed throughout the course of radiotherapy in this study’s patient population emphasises the need for future studies to substantiate these results and further establish whether such displacement has a clinical impact on the CTV and its coverage. The higher frequency of intracranial (89 %) versus extracranial (7 %) displacement could be attributed to the standard presence of thermoplastic immobilisation masks worn by patients during radiotherapy which may physically inhibit external movement of the bone flap.

Though this study only focused on proving and recording instances of bone flap displacement over the course of radiotherapy treatments, the impacts of such movement has been theorised in order to demonstrate the importance in investigating bone flap displacement further. As shown in Fig. 2, it is theorised that adapting the CTV margin or adjusting the PTV margin to accommodate changes from extracranial or intracranial bone flap displacement is essential for maintaining adequate target area coverage and achieving successful therapeutic outcomes. In cases of extracranial displacement failure to adapt the CTV or for PTV margins to accommodate the altered external bone position may result in underdosage of the target area. Similarly, the absence of CTV adaptation or adequate adjustments in PTV margins to address intracranial displacement could potentially result in radiation of healthy tissue due to the bone’s internal movement compressing or displacing the actual target area. These potential implications currently lack empirical evidence due to limited published bone flap displacement research. Therefore, the documented frequent presence of displacement found in this study proves that, with the existence of bone flap displacement, there is a need for future research to better understand the potential clinical implications of bone flap displacement occurring during radiotherapy treatments in order to ensure the most optimised treatment planning process possible.

Regarding the three risk factors investigated, only bone flap fixation technique was established as statistically significant with displacement being slightly greater in the group who received sutures than those who received 1–3 or 4–5 fixation plates. This finding is particularly significant for proton therapy compared to photon radiation as protons are notably more sensitive to high-density materials like fixation plates [10], [11]. High-density materials can cause the production of artifacts in the imaging process, leading to errors in the calculated proton ranges and negatively impacting the accuracy of dose planning and delivery [12]. If future studies find displacement to indeed have a clinical impact on CTV, a dilemma in proton treatment arises; multiple fixation plates were linked to the least amount of displacement in this study, but low-density sutures are best for limiting the production of artifacts. This conflict may result in patient selection for proton treatment being dependent on the fixation technique used, and a balance will have to be achieved through simultaneously limiting displacement, if proven to have a clinical impact, as well as the production of artifacts in order to achieve an overall optimal treatment outcome.

There are limitations regarding this study’s findings. Firstly, the generalisability is limited owing to it being a single institutional, retrospective study with a limited number of patients. Additionally, patients were only included if they had a clearly visible bone flap on the planning CT and CBCTs, making them not representative of all brain tumour patients receiving post-operative radiotherapy. Therefore, this study’s results should be verified in a larger prospective study in which displacement and effect of risk factors can be better established across a more representative population. Secondly, there was a possibility of minor discrepancies from human error to arise during the registration of CT images or the measuring of displacement. However, to minimise the creation and impact of such errors on the results, all measurements were conducted by a single individual (YW), limiting inter-observer variability to ensure the most consistent recording of displacement values possible. To confirm the reliability of one person making all the measurements, consistency in measurements was assessed by having two additional observers (NB, DH) independently repeat all displacement measurements for two patients. The high ICC values obtained from comparing the three sets of measurements for these two patients strongly indicate that all the measurements made by YW for the other patients are similarly reliable and accurate. Finally, the applicability of this study’s findings is limited as it solely determined and measured the presence of displacement rather than also exploring the subsequent impact of displacement on target volume margins, treatment planning, and dose. As only CBCTs were available for the included patients after every treatment fraction instead of higher quality CTs, it was not possible to accurately calculate the dosimetric impact of the displacement. Therefore, the next avenue for future studies is determining changes in volume of the resection cavity associated with extracranial and intracranial displacement, investigating the specific impact of this on the CTV, and evaluating whether these changes on resection cavity volume and CTV margins could affect overall dose coverage and delivery. To accurately measure such changes to the resection cavity and subsequent CTV and PTV margins, MRIs and CTs should be used instead of the CBCTs within this study as they provide better imaging to measure the impact of bone flap movement on treatment plans which is not possible to calculate with CBCTs alone.

To conclude, bone flap displacement during radiotherapy treatment was present in all 25 patients, demonstrating that the bone flaps failed to remain a fixed entity as is currently assumed when cropping CTVs of brain tumours to the bone. The displacement was found to not be dependent on time of day or time between surgery and radiotherapy. The fixation technique can influence bone displacement with more fixation plates resulting in less overall movement than sutures. The confirmed high frequency of displacement found necessitates an assessment of its clinical implications, particularly its overall potential impact on the resection cavity, target volumes, and dose coverage. If a significant impact on the CTV and resection cavity volume is discovered, displacement should be considered a factor requiring consideration during radiotherapy planning within the PTV margins.

**Waiver of Patient Consent** This is a retrospective case study. Patient consent has been waived by Ethic committee.

## Declaration of competing interest

The authors declare that they have no known competing financial interests or personal relationships that could have appeared to influence the work reported in this paper. This publication is part of the project “Making radiotherapy sustainable” with project number 10070012010002 of the Highly Specialised Care & Research programme (TZO programme) which is (partly) financed by the Netherlands Organisation for Health Research and Development (ZonMw).
